# Domestic cats (*Felis catus*) prefer freely available food over food that requires effort

**DOI:** 10.1007/s10071-021-01530-3

**Published:** 2021-07-26

**Authors:** Mikel M. Delgado, Brandon Sang Gyu Han, Melissa J. Bain

**Affiliations:** Department of Medicine and Epidemiology, School of Veterinary Medicine, University of California, Shields Ave, 2108 Tupper Hall, Davis, CA 95616-5270 USA

**Keywords:** Cats, Contrafreeloading, Activity, Animal welfare, Environmental enrichment

## Abstract

Contrafreeloading is the willingness of animals to work for food when equivalent food is freely available. This behavior is observed in laboratory, domesticated, and captive animals. However, previous research found that six laboratory cats failed to contrafreeload. We hypothesized that cats would contrafreeload in the home environment when given a choice between a food puzzle and a tray of similar size and shape. We also hypothesized that more active cats would be more likely to contrafreeload. We assessed the behavior of 17 neutered, indoor domestic cats (*Felis catus*) when presented with both a food puzzle and a tray across ten 30-min trials. Each cat wore an activity tracker, and all sessions were video recorded. Cats ate more food from the free feed tray than the puzzle (*t* (16) = 6.77, *p* < 0.001). Cats made more first choices to approach and eat from the tray. There was no relationship between activity and contrafreeloading, and there was no effect of sex, age, or previous food puzzle experience on contrafreeloading. Our results suggest that cats do not show strong tendencies to contrafreeload in the home environment, although some cats (*N* = 4) ate most food offered in the puzzle or showed weak contrafreeloading tendencies (*N* = 5). Eight cats did not contrafreeload. Cats who consumed more food from the puzzle, consumed more food in general, suggesting a relationship between hunger and effort. Further research is required to understand why domestic cats, unlike other tested species, do not show a strong preference to work for food.

**Supplementary Information:**

The online version contains supplementary material available at 10.1007/s10071-021-01530-3.

## Introduction

Foraging is a natural behavior: most animals in the wild must forage in some way to survive, whether by searching or ambush (Huey and Pianka 1981; Stephens and Krebs 1986). Domesticated and captive animals often are encouraged to engage in foraging behaviors as a form of enrichment. This may take the form of providing opportunities for animals to work for food, for example by hiding food in a substrate such as straw that can be foraged (e.g., domesticated pigs and hamadryas baboons, de Jonge et al. 2008; Jones and Pillay 2004); providing enrichment that allows animals to extract seeds from holes (parrots, Coulton et al. 1997); scattering food in enclosures (Malayan sun bears, Schneider et al. 2014); through presentation of live prey (African lions and Sumatran tigers, Bashaw et al. 2003); and with puzzle feeders that require manipulation to extract food (Gray parrots, van Zeeland et al. 2013). One goal of feeding enrichment is to adjust captive animals’ time budgets to more closely mimic those of freely living animals.

When tested, many animals will work for food when similar food is freely available, a phenomenon known as contrafreeloading (Inglis et al. 1997; Jensen 1963). A preference for contrafreeloading is indicated when an animal works for 50% or more of all obtained food (Osborne 1977). Contrafreeloading contradicts optimal foraging theory, which suggests that animals should maximize energy gained while minimizing costs (Stephens and Krebs 1986).

Some proposed reasons for why animals contrafreeload include boredom in captive environments (McGowan et al. 2010), reducing uncertainty while in captivity or uncertain environments (McGowan et al. 2010), stimulation-seeking (Inglis et al. 1997), and information gathering (Inglis et al. 2001). Specific environmental conditions can increase or decrease contrafreeloading behavior: for example, food deprivation and an increase in required effort to obtain food tend to decrease contrafreeloading whereas sensory deprivation increases it (Bean et al. 1999; Inglis et al. 1997).

Contrafreeloading is found in various captive and companion species, including wild or domesticated animals housed in sanctuaries, zoos, laboratories and homes. Species tested include humans, chimpanzees, macaques, chickens, jungle fowl, pigeons, grizzly bears, maned wolves, rats, giraffes, and pigs (de Jonge et al. 2008; Inglis et al. 1997; Jensen et al. 2002; McGowan et al. 2010; Sasson-Yenor and Powell 2019; Vasconcellos et al. 2012). Contrafreeloading is tested by providing animals with a simultaneous choice of freely available food and food that is acquired through some form of operant response (Inglis et al. 1997).

McGowan et al. found that captive-living, wild-born grizzly bears spent more time interacting with food boxes that required effort to obtain reinforcement than with freely available food (2010). A study of domestic pigs found that they tended to work for food even when the same type of food was available without effort (de Jonge et al. 2008). Contrafreeloading also has been observed in adult humans, although the effects were age-dependent, with younger participants preferring to press a lever for candy or cash rewards compared to older participants (Tarte 1981).

Contrary to other species tested, a study of six reproductively intact domestic cats found no evidence for contrafreeloading. Cats were first trained to offer paw touches to a switch plate for food on a continuous reinforcement schedule. When offered a choice between delivering an operant response or eating the same type of food that was freely available in a dish, cats consumed the freely available food first (Koffer and Coulson 1971). Based on this result, cats have been described as the only species to show no contrafreeloading (Inglis et al. 1997). However, the previous study had a relatively small sample size (*N* = 6), and the experiment was conducted in the laboratory environment with cats maintained at 85–90% of their free-feeding body weights. As previous studies have found that increased hunger reduces contrafreeloading (Inglis et al. 1997), food restriction may be just one factor that impacted cats’ willingness to work for food.

Based on studies of contrafreeloading and foraging behavior of wild-born species living in captivity, foraging enrichment is often recommended for companion animals, such as dogs, cats, and parrots (e.g., Dantas et al. 2016; Meehan and Mench 2007; Schipper et al. 2008). Although contrafreeloading has been studied in domesticated species such as swine and poultry (de Jonge et al. 2008; Lindqvist and Jensen 2008, 2009), there have been few studies of preferences to work for food in companion animals.

The goal of the present study was to further assess contrafreeloading in indoor-only domestic cats by testing normally fed cats in their home environments at multiple times. Contrafreeloading has previously been correlated with exploration (e.g., Bean et al. 1999) and may allow an individual to gather information about their environment (Inglis et al. 1997). For this reason, we also included a measure of each cat’s activity level to assess whether there is a relationship between activity and contrafreeloading. We predicted that most domestic cats would contrafreeload, preferring to eat food from a food puzzle designed for cats over freely available food from a tray of the same size and shape. Further, we predicted that cats with higher activity levels would show a stronger preference for contrafreeloading compared to cats with lower activity levels.

## Methods

All animal procedures were approved by the Animal Care and Use Committee at the University of California, Davis, under Protocol #21433. Data were collected between December 2019 and March 2020.

### Participants

Twenty domestic cats (11 male, 9 female) between 1 and 10 years old (average age: 5.1 years, SD: 3.1 years) were enrolled in the study. All cats were privately owned, neutered, living indoors, and free of any medical conditions. All cats were the only cat living in the home and regularly ate commercially available dry cat food. All cats were fed their regular diet for training and tests. Six of the 20 cats had previous experience with using a food puzzle.

### Equipment acclimation

Cats were acclimated to the experimental equipment for 4–12 days, depending on how quickly the cat adjusted. Owners of the participating cats were provided with a 13-g activity tracker (Fitbark Inc., Kansas City, MO) which was secured to a breakaway collar with zip ties and placed on each cat. The Fitbark^™^ is designed to detect movement via tri-axial accelerometer technology, and provides amalgamated activity levels for 1-min epochs when the device is worn. The device uses a proprietary point system to create an activity measure. Activity levels can be downloaded from the company’s website. Cat owners did not have access to the raw data or website unless requested after their participation was complete.

**Fig. 1:**
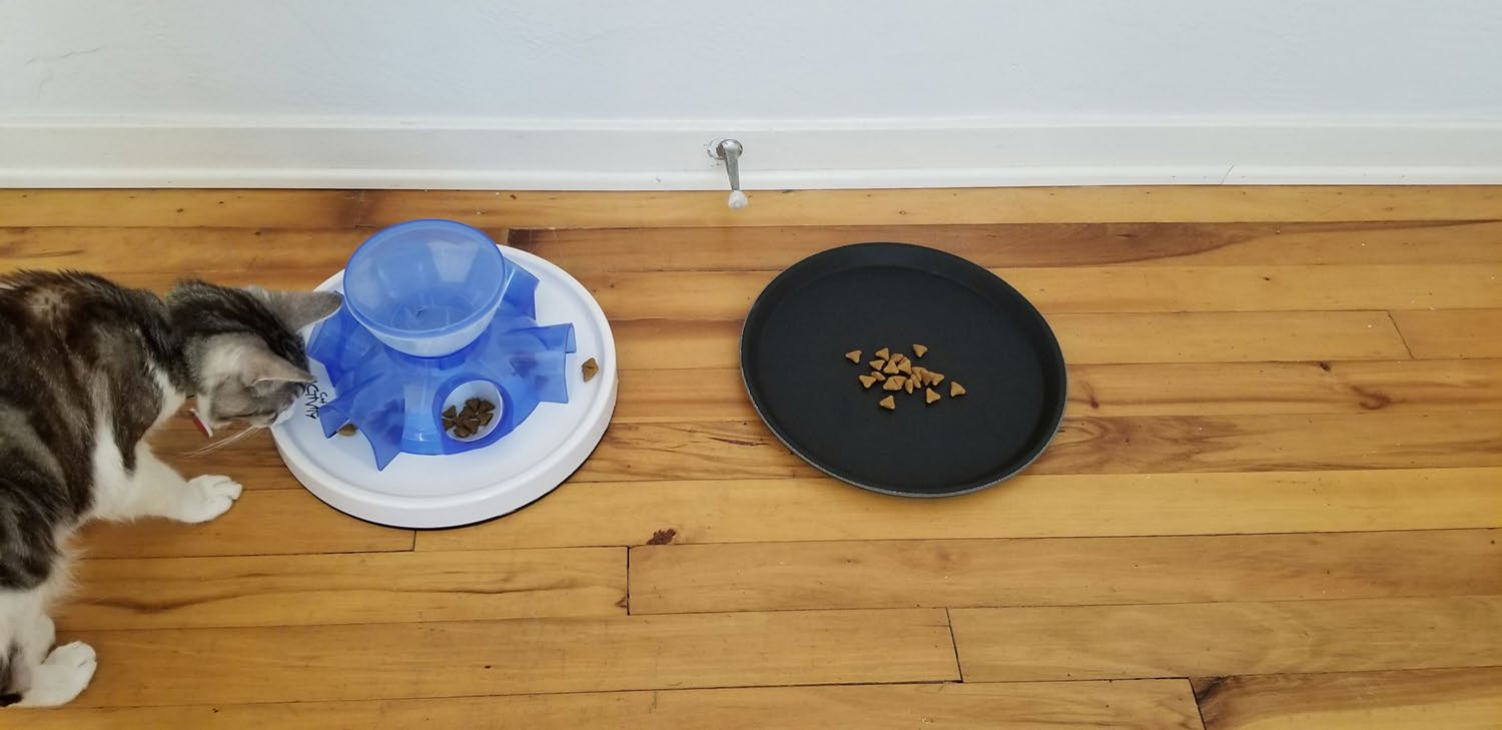
An example of the experimental set up with the food puzzle (Trixie Tunnel Feeder) and the tray of identical size/shape

Cats were simultaneously introduced to the Trixie Pet Tunnel Feeder food puzzle (Trixie Pet Products, Fort Worth, TX) using a protocol similar to that used by Naik et al., 2018. The puzzle requires cats to scoop food out of tunnels/compartments using their paws. Cats were also given a round plastic tray of an identical diameter as the puzzle as the control (Fig. 1). For the first 1–3 days, cats were given their regular amount of food on the plastic tray with an additional 25% of their daily intake provided in the puzzle. Cats who successfully consumed some food from the puzzle by that time were next provided 25% of their daily food rations in the puzzle and 75% on the tray for 1–3 days. The food was then split evenly between the tray and puzzle for 1–3 days, and on the last 1–3 days of training, the cats were provided 75% of their food in the puzzle, and 25% on the tray. Three cats (2 female, 1 male) who refused to consume any food from the puzzle during training were withdrawn from the study for their safety. Cats that successfully completed the training were then exposed to the testing protocol.

### Procedures

Cat owners were provided with a WyzeCam camera (Wyze Labs, Seattle, WA) and a secure digital (SD) card to record all trials. For each trial, cats were provided with an equal amount of food in the free food tray and the puzzle. Cat owners recorded two to four trials per day over the course of 3–4 days and were asked to wait at least 2 h between trials. Depending on the number of trials per day, the cat was given an appropriate percentage of their daily rations at each trial (e.g., if the owner was conducting four trials in a day, they gave their cat 25% of their daily rations for one trial).

Owners were provided with a standard food scale to weigh and record the amount of food offered in the puzzle and tray for each trial. Owners were also given a predetermined randomized sequence (random.org) for placing the puzzle on either the left or right side of the tray on each trial, to reduce any effects of side bias or preference. The cat was presented with the puzzle on the left or right an equal number of times. Owners were asked to place the puzzle and tray near each other, equidistant from the cat at the time of presentation. Owners were also asked to record the date and time each trial started, and the initial and final amount of food left on both the puzzle and tray. At the end of each trial, any remaining food was provided to the cat in their regular feeding dish. Cats were given 30 min to eat from the puzzle and tray for each trial, at which point both were removed and the recording was stopped.

### Behavior coding

Videos of trials were coded using BORIS (Behavioral Observation Research Interactive Software; (Friard and Gamba 2016)). We coded anytime the cat was eating from either the puzzle or tray, as well as any sniffing or touching of the puzzle or tray. Since discriminating between sniffing and eating was not always possible depending on the camera angle, we consolidated these interactions (eating, sniffing, and touching) into one measure for both the puzzle and the free food tray.

Five coders assessed the videos. All coders trained on a subset of 20–30 min of video, then were provided with additional training if needed. Overall inter-rater reliability of coders, as calculated by BORIS, was substantial, average Cohen’s kappa = 0.81 (range 0.73—0.91).

## Data analysis

To assess the contrafreeloading and activity of domestic cats, we used the following data: amount of food consumed from the puzzle and free food tray, time spent interacting with the puzzle and tray, proportion of first choice for the puzzle for interacting and eating during each trial, and the activity measure downloaded from the Fitbark™.

### ***Consumption from puzzle (P***_***total***_***) and tray (T***_***total***_***)***

The total food consumed from the puzzle and free food tray for each cat was calculated by adding the amount of food eaten from each for all trials.

### Strength of preference to feed and interact with puzzle (contrafreeloading)

The presence of contrafreeloading behavior was assessed using the following equations:

$${\text{CF}}_{\text{feeding}}= \frac{\mathrm{Food co}{\text{nsume}}\mathrm{d from puzzle }(P_{total})}{\mathrm{Food consumed from puzzle }(P_{total}) +\mathrm{ Food consumed from tray}(T_{total})}$$

$${\text{CF}}_{\text{time}}= \frac{\mathrm{Time spent at puzzle }(Tp)}{\mathrm{Time spent at puzzle }\left(Tp\right)+\mathrm{ Time spent at tray}(Tt)}$$

**Fig. 2:**
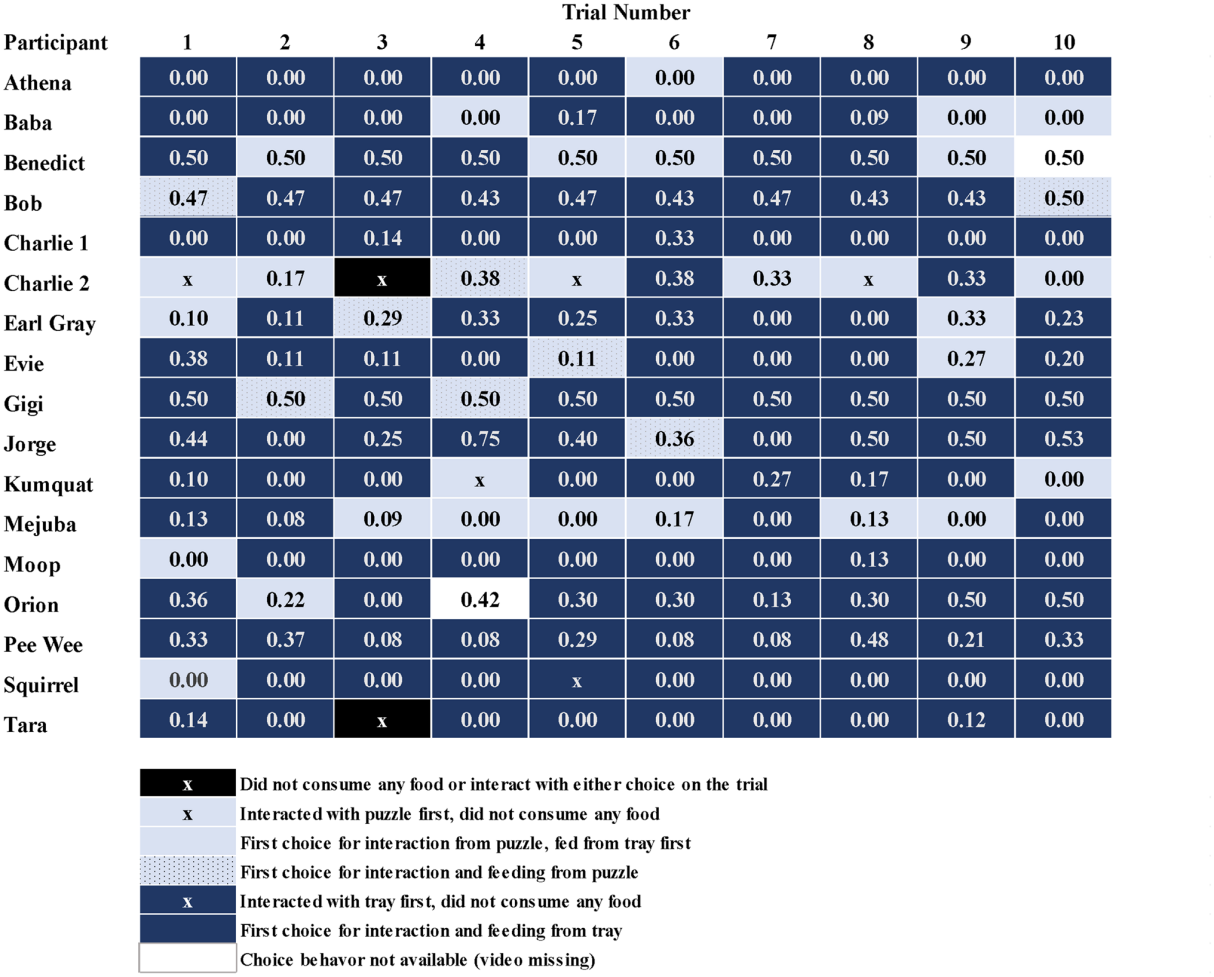
Data for each cat for each trial of the study. The figure depicts the cats’ first choices for interacting or feeding, and presents the data for CF_feeding_ for each trial. A value of 1 indicates complete preference for feeding from the puzzle, a value of 0.50 indicates consumption of the same amount of food from both, and a value of 0 indicates complete preference for feeding from the tray

To explore the consistency of each cat’s behavior across trials, we also calculated CF_feeding_ for each trial for each cat, which is depicted in Fig. 2. If the cat spent more time at, or consumed more food from, the puzzle than the free food tray, CF_feeding_ and CF_time_ will be greater than 0.5. A complete preference for the puzzle would result in values of 1. Equal time and consumption from both puzzle and tray would lead to values of 0.5. If the cat did not interact with or eat from the puzzle, CF_feeding_ and CF_time_ would be 0 (complete preference for the tray).

### ***First choice for puzzle (C***_***p***_***)***

C_p_ values were calculated by the following equation:

C_p_ = number of first choices made for the puzzle/total number of sessions.

We calculated C_p_ by observing both the first interaction a cat had (IC_p_; e.g., sniffing or touching either the puzzle or free food tray) as well as for which source they chose to eat from first (EC_p_). If the cat immediately started eating, then his/her choice (puzzle or tray) was marked as both the first choice for interacting and eating. C_p_ represents the proportion of first choices that were for the puzzle. Each cat’s choice behavior for each trial is depicted in Fig. 2, and the values for IC_p_ and EC_p_ are in Table 1.

**Table 1:** Data for each cat who completed the study, including sex, age, total food consumed from the puzzle and tray, time spent at the puzzle and tray, strength of contrafreeloading, percent first choices for interacting and eating from the puzzle, average daily activity, and classification of contrafreeloading behavior (strong/yes/weak/no)

Cat	Sex	Age	*P*_*total*_ (g)	*T*_*total*_ (g)	*CF*_*feeding*_	*T*_*p*_ (s)	*T*_*t*_ (s)	*CF*_*time*_	*EC*_*p*_	*IC*_*p*_	Average activity	CF?
Athena	F	5	0	54	0	277	1038	0.21	0	0.1	4821	No
Baba	M	3	3	92	0.03	106	1535	0.06	0	0.3	4143	No
Benedict	M	10	83	83	0.50	2726	410	0.87	0	0.4	3746	Yes
Bob	M	10	67	80	0.46	4059	2074	0.66	0.2	0.2	3896	Yes
Charlie	M	6	4	58	0.06	204	1440	0.12	0	0	5147	No
Charlie2	F	8	12	29	0.29	769	810	0.49	0.1	0.7	4656	Weak
Earl Gray	M	2	27	96	0.22	1656	3156	0.34	0	0.3	10,122	Weak
Evie	F	3	13	74	0.15	481	993	0.33	0.1	0.2	3711	Weak
Gigi	M	3	77.5	77.5	0.50	1509	440	0.77	0.2	0.2	6306	Yes
Jorge	M	5	52	70	0.43	2865	1440	0.67	0.4	0.4	4286	Yes
Kumquat	F	1	5	67	0.07	342	2130	0.14	0	0.2	6723	No
Mejuba	M	10	7	96	0.07	96	1444	0.06	0	0.6	3632	No
Moop	F	3	1	50	0.02	24	1367	0.02	0	0.1	3843	No
Orion	M	3	35	70	0.33	977	1157	0.46	0	0.11	5548	Weak
Pee Wee	M	3	41	114	0.26	1727	2208	0.44	0	0	N/A	Weak
Squirrel	F	2	0	59	0	9	1761	0.01	0	0.1	5656	No
Tara	F	10	2	49.5	0.04	3	1930	0	0	0	3448	No

*P*_*total*_ total grams of food consumed from puzzle, *T*_*total*_ total grams of food consumed from tray, *CF*_*feeding*_ strength of feeding preference for puzzle (P_total_/[P_total_ + T_total_]), *T*_*p*_ time spent at puzzle across all trials (seconds), *T*_*t*_ time spent at tray across all trials (seconds), *CF*_*time*_ strength of time preference for puzzle (T_p_/[T_p_ + T_t_]), *EC*_*p*_ proportion of first choices for puzzle to eat, *IC*_*p*_ proportion of first choices for puzzle to interact, *Average activity* average daily Fitbark points, *CF* tendency to contrafreeload

### Statistical analyses

All data were analyzed using SAS University Edition (SAS Institute Inc, Cary, NC). To assess the potential effects of individual differences, we used general linear models to determine if there were any effects of sex, age, and previous food puzzle experience on CF_feeding_ and CF_time_. We used a paired *t* test to compare the amount of food eaten from the puzzle to that eaten from the free food tray by each cat. Finally, we used correlation tests to assess the relationships between activity and contrafreeloading (CF_feeding_ and CF_time_).

## Results

Due to equipment error, activity data were not collected for one cat (Pee Wee). In addition, for two cats (Orion and Benedict), video data were collected for only 9 out of 10 trials. All other participants had complete video data for all 10 trials. All data used for analyses are presented in Table 1 and the supplementary data file.

We used general linear models to assess the effects of sex, age, and prior food puzzle experience on the two measures of contrafreeloading, CF_feeding_ and CF_time_. The overall model for the effects of sex, age and experience on CF_feeding_ was not statistically significant (*F *(3,13) = 2.93, *p* = 0.07). One factor, sex, had a *p* value < 0.05, but this should be interpreted with caution since the overall model was not statistically significant. The overall model for the effects of sex, age and experience on CF_time_ was not statistically significant (*F *(3,13) = 1.72, *p* = 0.21). None of the individual factors had a *p* value < 0.05.

We used a paired *t* test to compare the amount of food that cats consumed from the puzzle and the free food tray. Although most cats ate some food from both sources, the amount of food consumed from the tray was statistically higher than that eaten from the puzzle (*t *(16) = 6.77, *p* < 0.001). Almost half of the cats (*N* = 8, 5 females) consumed less than 10% of the food offered to them from the puzzle, and two of those cats (both female) ate no food offered from the puzzle during sessions.

To test the hypothesis that activity levels were related to contrafreeloading, we compared CF_feeding_ and CF_time_ with the average daily activity of each participating cat. Since larger values indicate more expression of contrafreeloading, CF_feeding_ and CF_time_ should be positively correlated with activity if cats contrafreeload. There was no correlation between CF_feeding_ and activity, Spearman correlation coefficient *r *(16) = −0.09, *p* = 0.75. There was no correlation between CF_time_ and activity, Spearman correlation coefficient *r *(16) = 0.21, *p* = 0.44. As an additional exploratory analysis, we assessed the correlation between the amount of food eaten from the tray and food eaten from the puzzle. The results suggested a positive relationship between the two, Spearman correlation coefficient *r *(16) = 0.56, *p* = 0.02; cats who ate more food from the tray also tended to eat more food from the puzzle.

Based on these results, we would classify four cats as willing to contrafreeload, five cats as weakly contrafreeloading, and eight cats as freeloaders (Table 1). Since no cats consumed more food from the puzzle than the free food tray, we would not classify any cats as strong contrafreeloaders.

## Discussion

In this study, we tested for the first time whether domestic cats living in homes would contrafreeload, as has been demonstrated in several captive and domesticated species. We did not find strong evidence for contrafreeloading; instead, cats preferred to eat the food that was freely available with no required additional effort. This was true when looking at both the overall population of cats, and the behavior of individual cats across trials.

We were unable to identify specific individual traits (sex, age and previous experience with a food puzzle) that predicted whether cats would contrafreeload. The four cats who appeared to be contrafreeloaders had one trait in common; they ate most of the food available to them during trials. In fact, the strongest predictor of amount of food eaten from the puzzle was the amount of food eaten from the tray.

The unanswered question is why cats, among multiple species tested, appear to be the only one that does not reliably contrafreeload. This tendency appears to contradict the fact that cats naturally work for food by hunting and will stop eating to hunt additional prey (Adamec 1976; Leyhausen 1979). Some differences in contrafreeloading tendencies among species have been attributed to domestication, such as in one study where white leghorn layers contrafreeloaded less than their ancestral species, jungle fowl (Jensen et al. 2002). Possible explanations included selection for allocation of resources toward reproduction and individual growth, and less need for information gathering when food sources are stable.

The cats in our study were all spayed or neutered and housed indoors only, which might have impacted their behavior. Spaying and neutering decreases the metabolic rate (Fettman et al. 1997) and the physical activity of female cats (Belsito et al. 2009), while simultaneously increasing cats’ food intake (Alexander et al. 2011; Wei et al. 2014). However, the previous study that failed to find evidence of contrafreeloading in cats only included reproductively intact cats (Koffer and Coulson 1971). To date, no studies have directly compared the activity patterns of indoor cats with those who have outdoor access. Thus, it would be premature to predict different results from reproductively intact or outdoor cats.

We also found no relationship between activity and contrafreeloading behavior. This result supports a previous study that found no change in activity level after introducing food puzzles to cats (Naik et al. 2018). Another study found that when two laboratory cats were required to offer an increasing number of touches to a switchplate in exchange for free access to food, they decreased the number of meals per day, and consumed more food at each feeding period (Collier et al. 1997). Studies of free-roaming feral cats suggest they spend almost 90% of their time inactive, with < 1% of their time spent hunting (Hernandez et al. 2018). In general, cats appear to conserve energy to the greatest extent possible, minimizing the amount of time and effort required to meet their caloric requirements, whether by hunting or engaging with enrichment devices in homes.

Our conclusion that cats prefer freely available food over that which requires effort is limited by some aspects of our study, such as a relatively small sample size. It is possible that the appearance of the food puzzle hindered consumption, as food was more visible in the tray. However, the cover of the puzzle was translucent, and several of the compartments face upward, such that the food would be visible from above. Although the novelty or shape of the food puzzle could have been a deterrent to the cats, we controlled for this by presenting all cats with a novel tray of the same shape and size simultaneously. All cats ate from both the puzzle and the tray during the training period, so we do not believe that appearance, novelty, or aversion to the device itself can explain the failure to contrafreeload.

We also did not control for food intake or hunger, as we did not want to increase the stress levels of cats, who can be sensitive to changes in feeding regimens (Stella et al. 2011). Food restriction can also reduce contrafreeloading (Inglis et al. 1997), so we did not want food withheld from cats longer than they were accustomed to. Since the cats ate a large percentage of the freely available food during trials, we cannot blame the failure to contrafreeload on a lack of interest in food. However, as we only gave cats access to the tray and puzzle for 30 min per trial, it is possible that cats would have engaged more with the puzzle if it continued to be available throughout the day.

Interpretation of our results may be dependent on how contrafreeloading is defined. Sometimes contrafreeloading is defined as when an animal will work for any food in the presence of freely available food, whereas some consider it a *preference* to work for food (Inglis et al., Osborne et al.). Most cats in our study did eat some food from the puzzle but none ate more food from the puzzle than the tray. Thus, although we have evidence for some willingness to work for food when freely available food is present (weak contrafreeloading), there is no evidence that cats preferred to work for food.

Of the species who have been tested for contrafreeloading, few predatory species (chimps, humans, and cats) are included, and most species tested are foragers who use extended search to acquire food (e.g., pigeons, rats, gerbils, Inglis et al. 1997). Sit-and-wait predation is considered low cost and is a common hunting style among felids (Williams et al. 2014). A full discussion of predatory energetics is beyond the scope of this manuscript, but contrafreeloading, which provides information about the quality of food patches, is expected to be weaker in species that do not engage in prolonged search (Inglis et al. 1997). Future studies should investigate whether foraging style is an important factor in contrafreeloading tendencies, and whether energy conservation is influential.

Like other studies, we did not find a statistically significant effect of sex on contrafreeloading (Lindqvist and Jensen 2008; Vasconcellos et al. 2012). However, in the current study, the four cats who did show a tendency to contrafreeload were all males, and the two cats who did not eat any food from the puzzle were both females. Male cats are more prone to obesity (Lund et al. 2005) and may be more food motivated in general. Our findings suggest the sex of the cat should be considered in future studies.

Future research can further explore contrafreeloading in cats by introducing different types of food puzzles or operant behaviors necessary for obtaining food. Changing the value of the food offered may also increase contrafreeloading, as novelty of food items increases the level of reinforcement (Inglis et al. 1997). The effects of foraging enrichment on cat welfare and health indicators should also be assessed. A presentation of case studies found positive effects of food puzzles for domestic cats, such as weight reduction and an improvement of behavioral health (Dantas et al. 2016), but puzzles do not appear to increase overall activity levels (Naik et al. 2018).

Understanding contrafreeloading is important for captive and domestic animal welfare as foraging enrichment is a frequently used tool to provide choice and mental stimulation. The effects of such enrichment on the behavior of captive animals are rarely tested. For domestic cats, the provision of foraging enrichment may depend on the needs and food motivation of the individual animal, and may be best introduced as a choice to enhance welfare, as foraging enrichment has been suggested to do in other species (e.g., Tarou and Bashaw 2007).

## Supplementary Information

Below is the link to the electronic supplementary material.

Supplementary file1 (XLSX 37 KB)

## Data Availability

Data are available to download at https://doi.org/10.25338/B8GG92.
